# Variants rs2200733 and rs6843082 Show Different Associations in Asian and Non-Asian Populations With Ischemic Stroke

**DOI:** 10.3389/fgene.2022.905560

**Published:** 2022-08-18

**Authors:** Dongsen Wang, Xuemei Hu, Xue Yang, Mingfeng Yang, Qingjian Wu

**Affiliations:** ^1^ Clinical Medical College of Jining Medical University, Jining, China; ^2^ Department of Emergency, Jining No. 1 People’s Hospital, Jining, China; ^3^ Second Affiliated Hospital, Key Laboratory of Cerebral Microcirculation in Universities of Shandong, Brain Science Institute, Shandong First Medical University and Shandong Academy of Medical Sciences, Taian, China

**Keywords:** ischemic stroke, genome-wide association study, rs2200733, rs6843082, population

## Abstract

A previous genome-wide association study (GWAS) has reported that variants rs2200733 and rs6843082 in the paired-like homeodomain transcription factor 2 (*PITX2*) gene may be one of the risk factors for ischemic stroke (IS) in European populations. However, more recently, studies in Asia have reported that rs2200733 and rs6843082 are only weakly or not associated with increased risk of IS. This difference may be caused by the sample size and genetic heterogeneity of rs2200733 and rs6843082 among different races. For this study, we selected eight articles with nine studies from the PubMed and Embase databases, including five articles from Asian and three articles from non-Asian, to evaluate the risk of IS caused by rs2200733 and rs6843082. Then, we investigated rs2200733 and rs6843082 single-nucleotide polymorphisms (SNPs) by analysis using allele, recessive, dominant, and additive models. We identified that rs2200733 and rs6843082 are weakly significantly associated with IS for the allele model (*p* = 0.8), recessive model (*p* = 0.8), dominant model (*p* = 0.49), and additive model (*p* = 0.76) in a pooled population. Next, we performed a subgroup analysis of the population, the result of which showed that rs2200733 and rs6843082 covey genetic risk for IS in a non-Asian population, but not in an Asian population. In conclusion, our analysis shows that the effect of *PITX2* rs2200733 and rs6843082 SNPs on IS risk in Asia is inconsistent with the effect observed in European IS cohorts.

## Introduction

Ischemic stroke (IS) is the second leading cause of death worldwide and the main leading cause of intellectual disability in adults (Orellana-Urzua et al., 2020). The pathogenesis of IS has been studied using genome-wide association studies (GWAS), which provide a crucial direction for studying the genetic mechanism of IS (Chauhan and Debette, 2016; Liu et al., 2019b; Wei et al., 2019). In 2008, the *PITX2* rs2200733 and rs10033464 variants were identified as significant contributors to IS in a European population (*p* = 2.18 × 10^–10^) (Gretarsdottir et al., 2008). However, a series of subsequent studies failed to replicate those results.

In 2009, Shi et al. analyzed 383 patients with atrial fibrillation (AF) *versus* (*vs*.) 851 patients without AF and 811 patients with IS *vs*. 688 patients without IS, all of Chinese. After analysis, rs2200733 was meaningfully correlated with AF (*p* = 4.1 × 10^–12^) but not IS in this Chinese population (Shi et al., 2009).

In 2012, Bertrand et al. analyzed 3548 patients with stroke *vs*. 5972 patients without stroke and then replicated their result in 5859 patients with stroke *vs*. 6281 patients without stroke, all of European ancestry. Their results showed that both rs2200733 and rs1906599 were associated with IS (OR = 1.32) (International Stroke Genetics Consortium et al., 2012). Their study again identified a significant association between rs2200733 and IS.

In 2022, Zhao et al. analyzed 476 patients with IS *vs*. 501 control individuals, all Chinese (Zhao et al., 2022). Their analysis found no meaningful association between rs6843082 and IS (*p* = 0.448).

In summary, previous studies have reported different results as to whether rs2200733 and rs6843082 increase susceptibility to IS. It is not clear whether the two SNPs (rs2200733 and rs6843082) are related to IS susceptibility. In this study, we further evaluate whether these two SNPs (rs2200733 and rs68430828) increase the risk of IS using nine studies from eight articles.

## Materials and Methods

### Literature Search

The relevant literature was searched in PubMed (http://www.ncbi.nlm.nih.gov/pubmed) and Embase (https://www.embase.com/) databases. We filtered all relevant studies based on the keywords “Stroke,” “PITX2,” “rs2200733,” and “rs6843082.” The literature search was completed by 10 March 2022. In the following paragraph, we describe the criteria for inclusion.

### Inclusion Criteria

The inclusion criteria for our meta-analysis were as follows: (1) the study used a case–control design, (2) the study evaluated whether the two SNPs (rs2200733 and rs6843082) are risk factors for IS, (3) the study provided a clear and definite number of genotypes or alleles or enough data to calculate these numbers, and (4) the study provided an explicit odds ratio (OR) and 95% confidence interval (CI) or sufficient data to calculate the OR and 95% CI. All studies that did not meet the inclusion criteria were eliminated.

### Data Extraction

For each study that met the inclusion criteria, we extracted the following information: (1) first author, (2) year of publication, (3) race of the study subjects, (4) number of cases and controls, and (5) quantity of rs2200733 and rs6843082 genotypes in cases and controls. The full results are shown in Table 1.

**TABLE 1:** Characteristics of studies included in the meta-analysis.

SNPs	Study	Population	Case	Control	Case genotypes				Control genotypes			
					AA	AG	GG	AA		AG	GG
rs2200733	Gretarsdottir et al. (2008)	European	29474	6222	514	6754	22206	71		1189	4962
	Shi et al. (2009)	Chinese	811	688	200	405	206	180		344	164
	Bevan et al. (2012)	European	5859	6281	NR	NR	NR	NR		NR	NR
	Cao et al. (2013)	Chinese	1388	1629	311	692	385	342		809	478
	Su et al. (2015)	Chinese	816	816	194	417	205	191		408	217
rs6843082	Su et al. (2015)	Chinese	816	816	49	305	462	60		316	440
	Wu et al. (2015)	Chinese	167	176	12	66	89	20		78	78
	Ferreira et al. (2019)	Brazilian	240	285	128	95	17	140		120	25
	Zhao et al. (2022)	Chinese	476	501	34	187	255	40		203	258

SNP, single-nucleotide polymorphisms; NA, not publicly available; IS, ischemic stroke.

### Genetic Model

We used four common genetic models for this meta-analysis, including the allele model (A *vs*. G), recessive model (AA *vs*. AG+GG), dominant model (AA+AG *vs*. GG), and additive model (AA *vs*. GG). These results are helpful to evaluate the susceptibility to IS with the two SNPs (rs2200733 and rs6843082): A allele *vs*. G allele.

### Hardy–Weinberg Equilibrium

The HWE of the two SNPs (rs2200733 and rs6843082) in IS cases and the control group were analyzed using the Chi-square test. The relationship between the two SNPs (rs2200733 and rs6843082) and IS was analyzed using four gene models: allele model (A *vs*. G), recessive model (AA *vs*. AG+GG), dominant model (AA+AG *vs*. GG), and additive model (AA *vs*. GG). We performed all relevant Chi-square tests using the R program (http://www.r-project.org/).

### Heterogeneity Test

First, we extracted the summary statistical information corresponding to the two SNPs (rs2200733 and rs6843082) in the above study. Then, Cochran’s Q test and I^2^ = [Q—(k—1)]/Q × 100% (Liu et al., 2017) were used to analyze the heterogeneity of the two SNPs (rs2200733 and rs6843082) among these datasets. The Q statistic approximately follows a χ^2^ distribution with k-1 degrees of freedom (k stands for the number of studies for analysis). When the P value from Cochran’s Q statistic <0.1 and the I^2^ value from Cochran’s Q statistic >50%, the data showed considerable heterogeneity (Hu et al., 2017; Liu et al., 2017).

### Meta-Analysis

In Cochran’s Q statistic, if *p* < 0.05 or I^2^ >50%, it indicated that there was heterogeneity between studies, and a random-effect model (DerSimonian–Laird) was used to calculate the pooled OR. If not, we used a fixed-effect model (Mantel–Haenszel). All statistical methods in the meta-analysis were applied by program R (http://www.r-project.org/).

### Publication Bias Analyses

In this analysis, we used funnel plots to assess the possible publication bias. When there was no publication bias, the plot of the funnel was symmetrically inverted. Otherwise, it was an asymmetric inverted funnel (Liu et al., 2014). The asymmetry of the funnel plot was evaluated by the Egger test. We performed all statistical tests using the R program (http://www.r-project.org/).

## Results

### Comprehensive Literature Search

**FIGURE 1:**
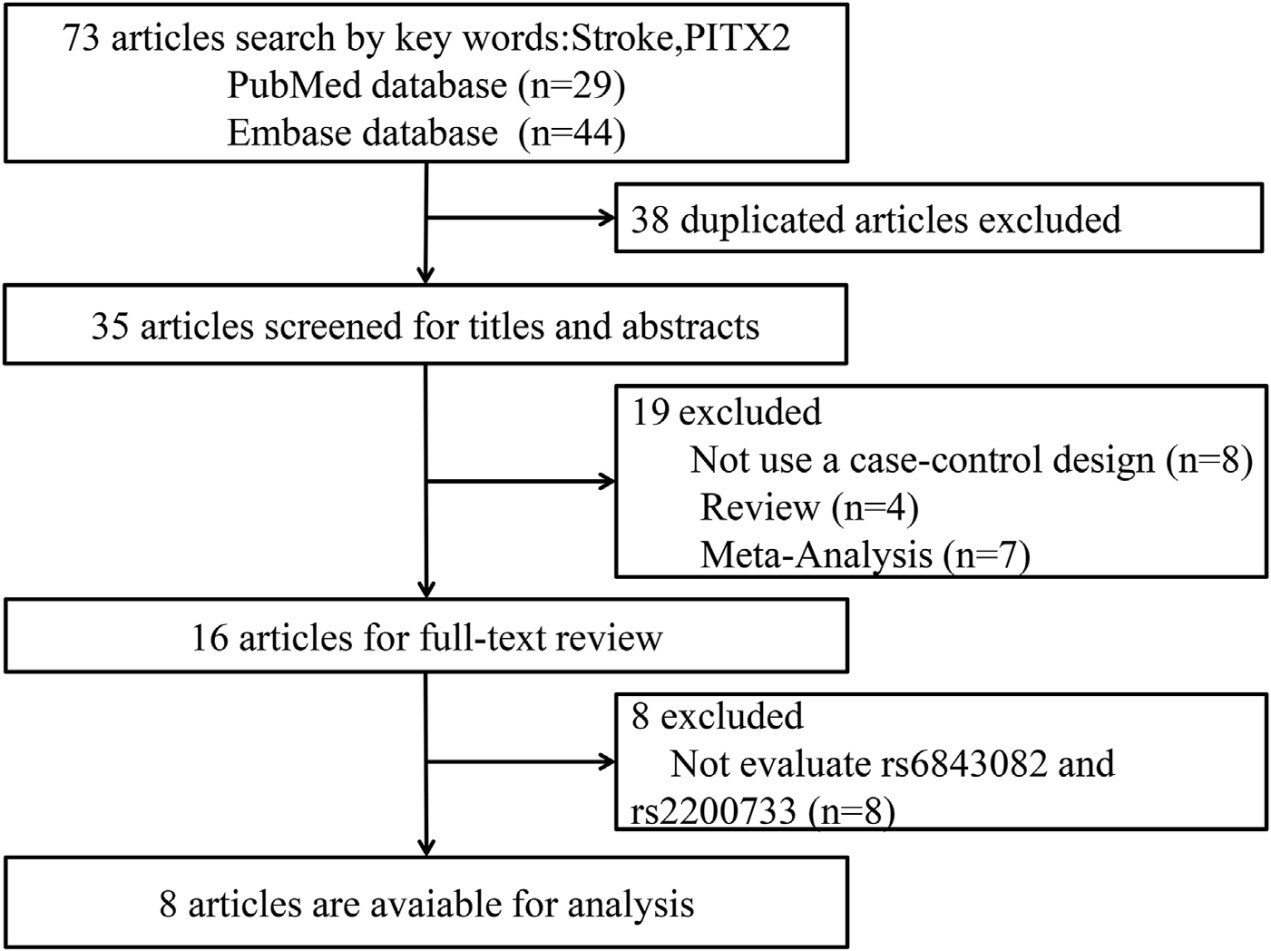
Flowchart of the selection of studies included in this meta-analysis.

We retrieved 29 articles from PubMed and 44 articles from the Embase database. Finally, eight articles (Gretarsdottir et al., 2008; Shi et al., 2009; Bevan et al., 2012; Cao et al., 2013; Su et al., 2015; Wu et al., 2015; Ferreira et al., 2019; Zhao et al., 2022), including nine studies, were chosen for meta-analysis by excluding overlapping studies. A total of 55,829 participants were included in this meta-analysis: 39,231 cases in the case group (38,348 cases with rs2200733 and 1699 cases with rs6843082) and 16,598 cases in the control group (15,636 cases with rs2200733 and 1778 cases with rs6843082). The study by Su et al. analyzed the two SNPs based on 1632 participants, and so the total number of case and controls overlapped. The specifics are shown in Figure 1. The primary features of the studies we included are presented in Table 1 **.**

### Linkage Disequilibrium

The rs2200733 and rs6843082 SNPs were located within 10 kb on *PITX2* gene (https://snipa.helmholtz-muenchen.de/snipa3/).

### Heterogeneity Test

The study of Bevan et al. was excluded from the dominant, recessive, and additive models. For this analysis, we observed no remarkable heterogeneity in the pooled population when using the four genetic models (Table 2).

**TABLE 2:** Analysis of four genetic models’ association of rs2200733 and rs6843082 with ischemic stroke.

Model	Asian		Non-Asian		Pooled population	
	OR (95%CI)	P	OR (95%CI)	P	OR (95%CI)	P
Allele (A *vs*. G)	0.92 (0.73–1.15)	0.45	1.03 (0.85–1.26)	0.74	0.98 (0.85–0.14)	0.80
Recessive (AA *vs*. AG+GG)	0.94 (0.69–1.28)	0.70	1.38 (0.81–2.35)	0.24	1.04 (0.79–1.36)	0.80
Dominant ( AA+AG *vs*. GG)	0.95 (0.72–1.26)	0.73	1.30 (0.93–1.81)	0.13	1.08 (0.87–1.33)	0.49
Additive ( AA *vs*. GG)	0.93 (0.67–1.30)	0.67	1.54 (0.84–2.82)	0.16	1.05 (0.78–1.40)	0.76

OR: odds ratio.

### Meta-Analysis With the Allele Model

**FIGURE 2:**
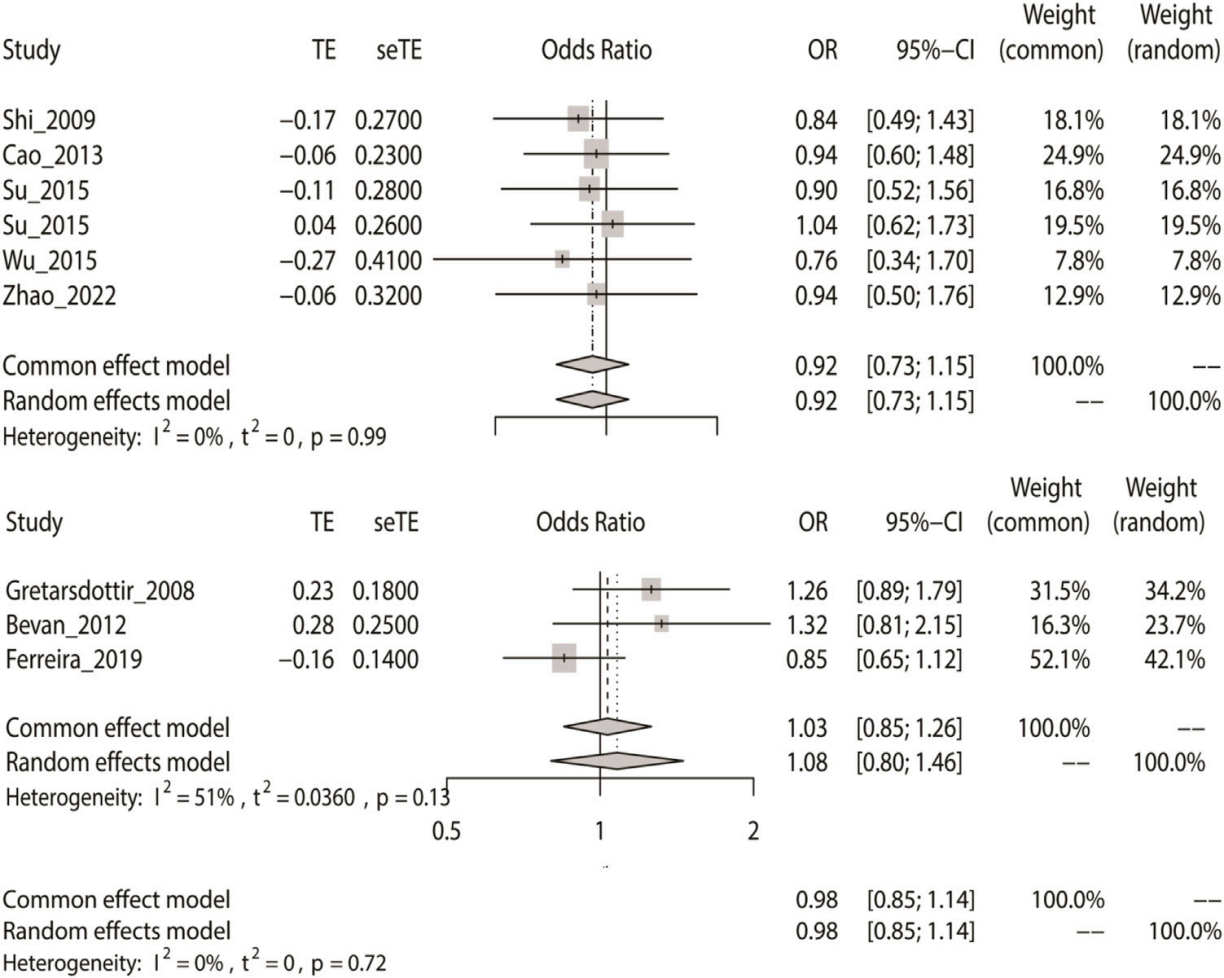
Fixed-effect meta-analysis of the allele model for rs2200733 and rs6843082 in the Asian, non-Asian, and pooled populations.

We computed the overall OR using a fixed-effect model in accordance with the outcomes of the heterogeneity test. The allele model tests showed that IS did not have a relationship with rs2200733 and rs6843082 in the Asian (*p* = 0.45), non-Asian (*p* = 0.74), and pooled populations (*p* = 0.80) (Table 2). Our results also showed that the two SNPs did not contribute to IS in Asian populations (OR = 0.92), but interestingly, the opposite results were seen in non-Asian populations, where both rs2200733 and rs6843082 were genetic risk factors for IS (OR = 1.03) (Figure 2).

### Meta-Analysis With the Recessive Model

**FIGURE 3:**
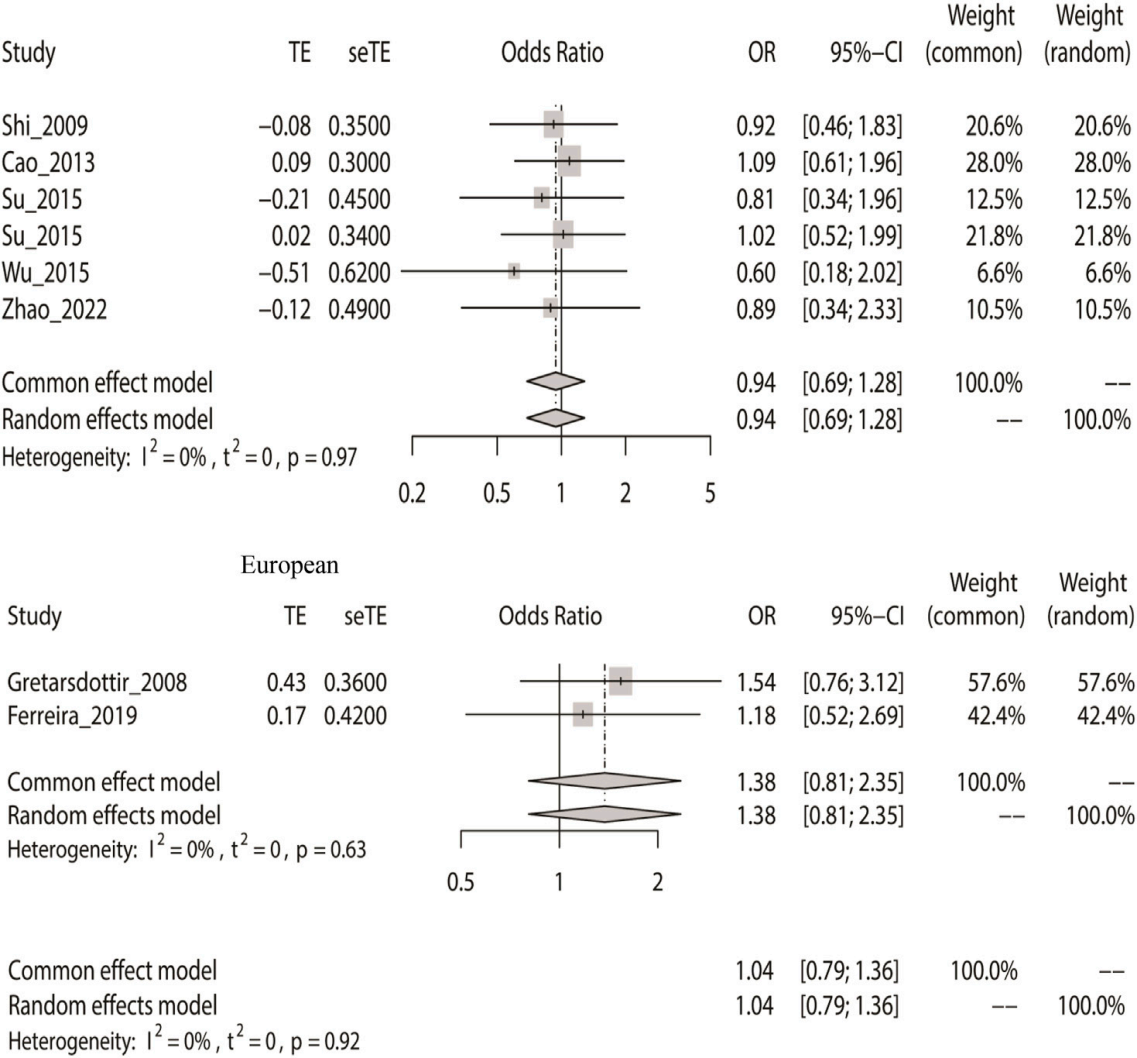
Fixed-effect meta-analysis of the recessive model for rs2200733 and rs6843082 in the Asian, non-Asian, and pooled populations.

Similarly, we calculated the overall OR using a fixed-effect model based on the recessive model. The recessive model indicated that rs2200733 and rs6843082 and IS in the Asian (*p* = 0.70), non-Asian (*p* = 0.24), and pooled population (*p* = 0.80) (Table 2) were not closely related. The two SNPs were not associated with IS in Asian populations (OR = 0.94). Conversely, rs2200733 and rs6843082 could increase the incidence of IS disease in non-Asian populations (OR = 1.38) (Figure 3).

### Meta-Analysis With the Dominant Model

**FIGURE 4:**
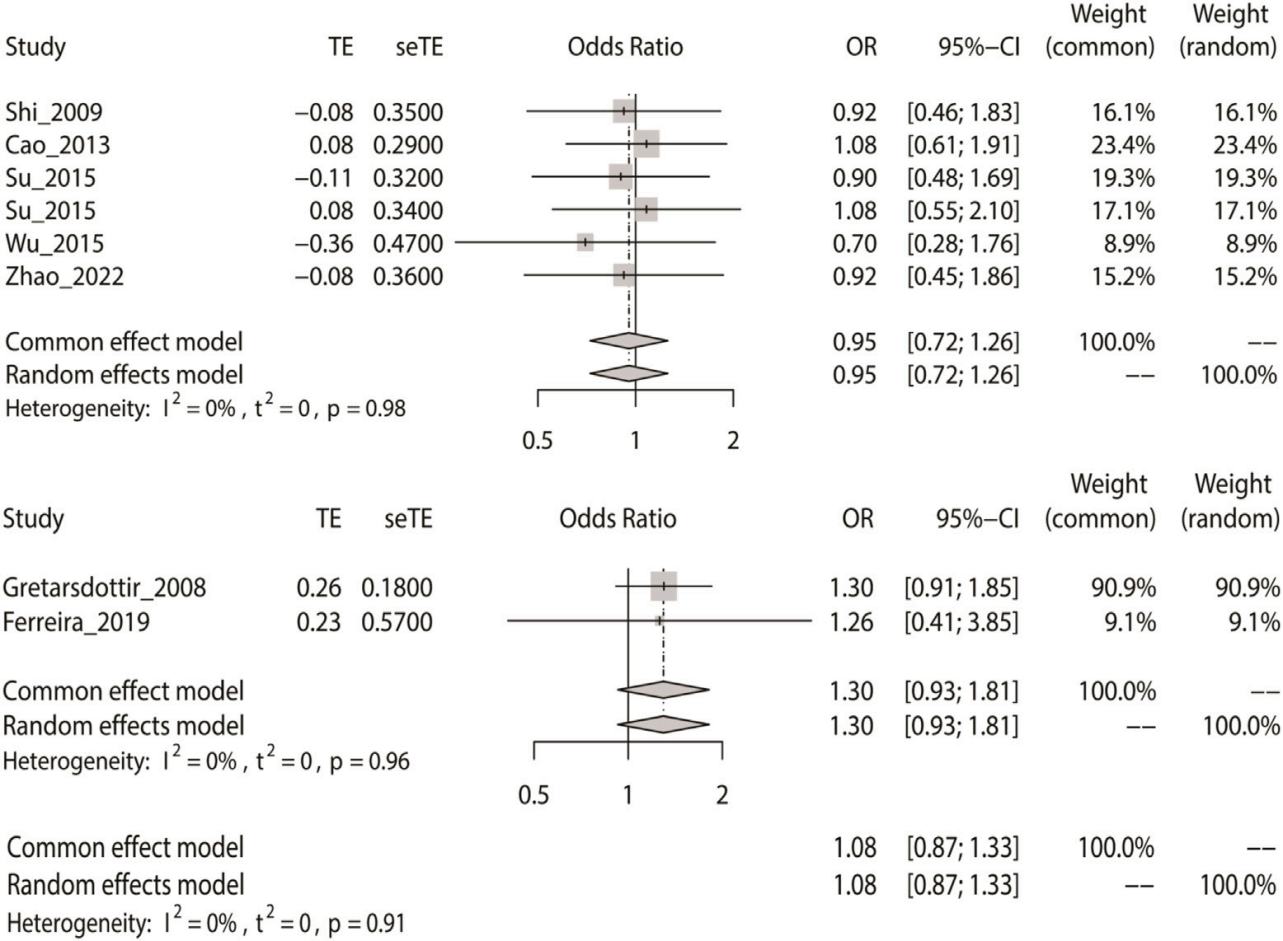
Fixed-effect meta-analysis of the dominant model for rs2200733 and rs6843082 in the Asian, non-Asian, and pooled populations.

Likewise, we calculated the overall OR using a fixed-effect model in accordance with the dominant model in the three groups. The dominant model showed that the two SNPs (rs2200733 and rs6843082) had no significant relationship with IS in Asian (*p* = 0.73), non-Asian (*p* = 0.13), and pooled (*p* = 0.49) populations (Table 2). The result of the subgroup analysis indicated that in the Asian population, the two SNPs were not genetic risk factors for IS (OR = 0.95); however, in the non-Asian population (OR = 1.30), the two SNPs were genetic risk factors for IS (OR = 1.08) (Figure 4).

### Meta-Analysis With the Additive Model

**FIGURE 5:**
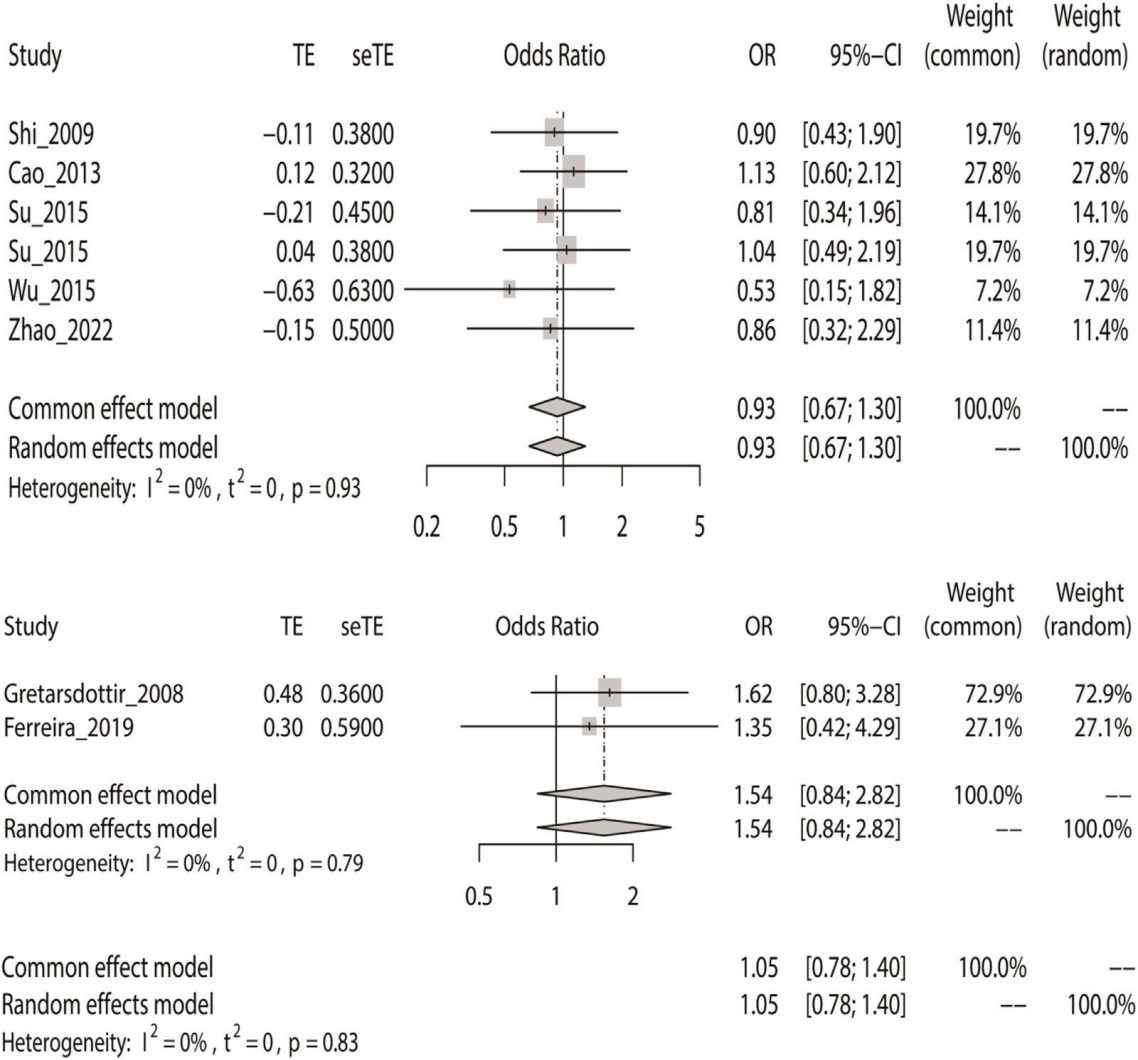
Fixed-effect meta-analysis of the additive model for rs2200733 and rs6843082 in the Asian, non-Asian, and pooled populations.

Finally, we used the fixed-effect model to calculate the overall OR based on the additive model, where IS had no meaningful relationship with the two SNPs (rs2200733 and rs6843082) in Asian (*p* = 0.67), non-Asian (*p* = 0.16), and pooled populations (*p* = 0.76) (Table 2). The results were the same as the three previous genetic models; the two SNPs were not associated with IS in the Asian population (OR = 0.93); however, rs2200733 and rs6843082 were associated with an increased incidence of IS disease in the non-Asian population (OR = 1.54) (Figure 5).

### Publication Bias Analysis

**FIGURE 6:**
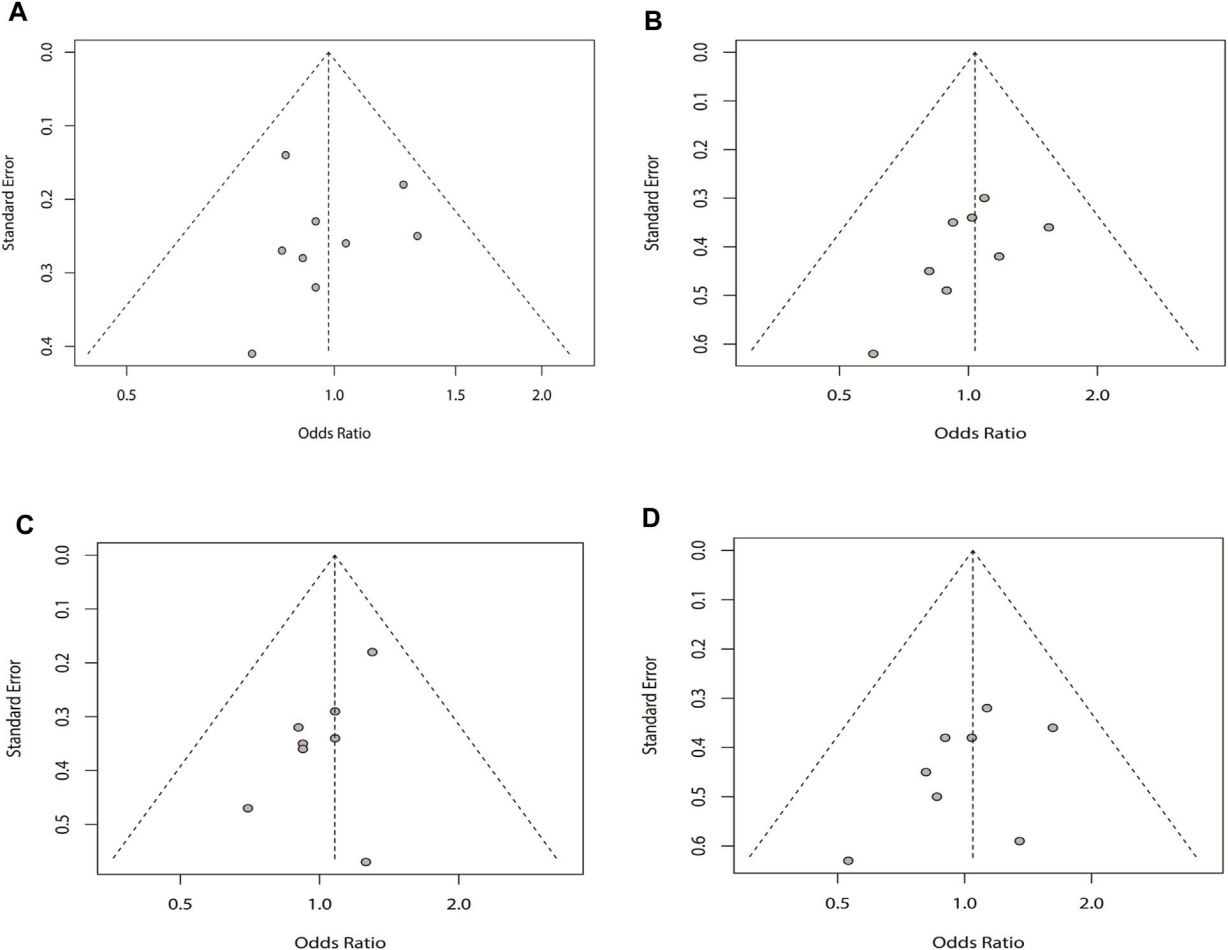
Sensitivity analysis of the four genetic models for rs2200733 and rs6843082 in the pooled population: **(A)** allele model for the two SNPs in the pooled population, **(B)** recessive model for the two SNPs in the pooled population, **(C)** dominant model for the two SNPs in the pooled population, and **(D)** additive model for the two SNPs in the pooled population.

The funnel plot and Egger’s test were applied to assess the existence of the potential publication bias in the four genetic models. There was no bias in the four plots, which were symmetrical inverted funnels. For the allele, recessive, dominant, and additive models, *p* = 0.943, 0.133, 0.053, and 0.204, respectively (Figure 6).

## Discussion

Previous GWAS studies have shown that rs2200733 and rs6843082 SNPs in *PITX2* are associated with genetic susceptibility to IS in European populations (Gretarsdottir et al., 2008). Subsequently, however, our results indicated that rs2200733 and rs6843082 conveyed no increased risk of IS. Overall, most studies have shown that the rs2200733 SNP in *PITX2* is associated with European IS, but five studies conducted in Chinese populations all concluded that the rs2200733 SNP was not associated with the IS risk. Meanwhile, three studies analyzed the association between rs2200733 and AF in the Chinese population, and the results suggested that the expression of rs2200733 had a potential genetic risk for AF, but not IS (Shi et al., 2009; Cao et al., 2013; Su et al., 2015).

In accordance with the analysis of the two SNPs (rs2200733 and rs6843082) in a pooled population, we can conclude that the G allele has low importance for the risk of IS. In the subgroup analysis, the results showed that the two SNPs had no correlation with the risk of IS in an Asian population, but the results in a non-Asian population showed a significant relevance with the risk of IS. These results suggest that the specific gene expression of that population and/or disease could be affected by genetic variation (Liu et al., 2019a). Therefore, two possibilities may lead to different associations between the two SNPs and human IS gene expression. The first factor is the racial difference, such as the genetic difference between Asian and non-Asian populations. For example, Gretarsdottir et al. indicated that rs2200733 has a strong association with IS (Gretarsdottir et al., 2008), but Cao et al. showed that rs2200733 has no association with any type of stroke (Cao et al., 2013). The second probability is that the disease condition has an effect on gene expression (Tammen et al., 2013; Maruthai et al., 2022). Haplotype association analysis by Su et al. showed that rs6843082 was significantly correlated with serum total cholesterol (TC) in IS patients in the additive and dominant models (Su et al., 2015). Moreover, for female individuals, the result of the recessive model also showed that rs2200733 was associated with high levels of TC, increasing the risk of IS (Su et al., 2015). The two SNPs (rs2200733 and rs6843082) are closely related to the *PITX2* gene on chromosome 4q25, which is associated with cardiac morphogenesis, particularly the differential identity of the left atrium from the right atrium and the growth of myocardial sleeves of pulmonary veins (Logan et al., 1998; Mommersteeg et al., 2007; Tessari et al., 2008). Therefore, the *PITX2* gene may be expressed during the development of the circulatory system and play a role in IS-related risk factors. Further studies are required to determine whether these two SNPs are risk factors for IS and provide a new direction for the treatment of IS.

Numerous studies on the relationship between *PITX2* and IS have produced conflicting results. So far, there is still no final and unanimous conclusion. Our analysis includes samples from multiple researchers; therefore, the results of our study may be more reliable than those of single studies. However, some potential limitations in our meta-analysis should be acknowledged. First, there was a small sample of GWAS and candidate gene studies, which may influence the pooled estimated value. Second, environmental factors, such as smoking and alcohol use, may affect the risk of IS, but some studies did not consider these risk factors. Third, different populations have different genetic susceptibilities both to the two SNPs (rs2200733 and rs6843082) and to IS, which can make the primary cause difficult to distinguish. IS is a disease that is effected by the interactions of multiple environmental and genetic factors (Dichgans, 2007; Cai et al., 2020), and so the influence of genes and environment on the pathogenesis of IS needs to be more deeply investigated.

## Data Availability

The original contributions presented in the study are included in the article/Supplementary Material; further inquiries can be directed to the corresponding authors.
